# Obesity-induced kidney injury is attenuated by amelioration of aberrant PHD2 activation in proximal tubules

**DOI:** 10.1038/srep36533

**Published:** 2016-11-09

**Authors:** Koji Futatsugi, Hirobumi Tokuyama, Shinsuke Shibata, Makiko Naitoh, Takeshi Kanda, Hitoshi Minakuchi, Shintaro Yamaguchi, Koichi Hayashi, Yoji Andrew Minamishima, Motoko Yanagita, Shu Wakino, Hiroshi Itoh

**Affiliations:** 1 Department of Internal Medicine, School of Medicine, Keio University, Tokyo, Japan; 2 Department of Physiology, School of Medicine, Keio University, Tokyo, Japan; 3 Department of Biochemistry, School of Medicine, Keio University, Tokyo, Japan; 4 Department of Nephrology, Graduate School of Medicine, Kyoto University, Kyoto, Japan

## Abstract

The involvement of tissue ischemia in obesity-induced kidney injury remains to be elucidated. Compared with low fat diet (LFD)-mice, high fat diet (HFD)-fed mice became obese with tubular enlargement, glomerulomegaly and peritubular capillary rarefaction, and exhibited both tubular and glomerular damages. In HFD-fed mice, despite the increase in renal pimonidazole-positive areas, the expressions of the hypoxia-responsive genes such as Prolyl-hydroxylase PHD2, a dominant oxygen sensor, and VEGFA were unchanged indicating impaired hypoxic response. Tamoxifen inducible proximal tubules (PT)-specific *Phd2* knockout (*Phd2-cKO*) mice and their littermate control mice (*Control*) were created and fed HFD or LFD. *Control* mice on HFD (*Control* HFD) exhibited renal damages and renal ischemia with impaired hypoxic response compared with those on LFD. After tamoxifen treatment, HFD-fed knockout mice (*Phd2-cKO HFD*) had increased peritubular capillaries and the increased expressions of hypoxia responsive genes compared to *Control* HFD mice. *Phd2-cKO HFD* also exhibited the mitigation of tubular damages, albuminuria and glomerulomegaly. In human PT cells, the increased expressions of hypoxia-inducible genes in hypoxic condition were attenuated by free fatty acids. Thus, aberrant hypoxic responses due to dysfunction of PHD2 caused both glomerular and tubular damages in HFD-induced obese mice. *Phd2*-inactivation provides a novel strategy against obesity-induced kidney injury.

Obesity is worldwide health problem with a variety of complications including hypertension, dyslipidemia, and abnormal glucose metabolism. Obesity is also considered as a major risk factor for chronic kidney disease (CKD)^1^. Renal structural and functional changes develop early during obesity whose lesions might affect those observed in the early phase of diabetic nephropathy, a leading cause of end-stage renal disease^2^,^3^. Previous studies of renal histological changes in obesity focused primarily on the glomerular area^4^. Obesity-related glomerulopathy has been well defined, characterized by glomerulomegaly with or without focal segmental glomerulosclerosis^4^. We recently reported that excess in fat intake causes obesity and renal injury in C57BL/6J mice, with not only enlarged glomeruli, but also enlarged and vacuolated tubules, particularly proximal tubules (PT)^5^. Other investigators also reported histological changes in the renal tubules in obesity and diabetes^6^,^7^.

Although previous studies reported that diabetes induced a hypoxic condition in the kidney, it has not been determined whether this condition is also evident in obesity^8^,^9^. PT cells are easily damaged by hypoxia because their volume accounts for more than 80% of the kidney cortex, to which oxygen is supplied only by the peritubular capillaries^10^. PT cells consume oxygen constantly, utilizing it for energy production to drive reabsorption and excretion of various substances. Accordingly, hypoxia, as a pathologic imbalance between oxygen supply and consumption, affects the PT areas. Therefore, hypertrophic PT cells in obese subjects might be readily susceptible to an inefficient oxygen supply, resulting in a hypoxic condition.

When there is an imbalance between oxygen demand and supply, cells activate hypoxic responses to protect themselves against the hypoxic environment. Among the molecules responsible for tissue hypoxic response, the transcription factor hypoxia-inducible factor (HIF), which is composed of HIF-1, HIF-2 and HIF-3, is the master regulator of the hypoxic responses. Although little is known about the cell type-specific functions of HIFs in response to ischemic kidney injury, many cellular adaptive processes are regulated by HIF-1^11^,^12^. Experiments on cells bearing inactivating mutations in the HIF pathway have emphasized the importance of HIF-1 on the regulation of genes involved in angiogenesis^13^. HIF-1 consists of α-subunit (HIF-1α) and β-subunit (HIF1-β/ARNT), and HIF-1α proteins are targeted for protein degradation by prolyl-hydroxylases (PHDs)^14^. PHDs function as tissue oxygen sensor molecules by regulating the stability of HIFα proteins in an oxygen-dependent manner. In normal oxygen availability, *p*rolyl hydroxylation of HIFα by PHDs enables its association with the von Hippel-Lindau tumor suppressor E3 ubiquitin ligase complex, leading to the degradation of HIFα via the ubiquitin-proteasome pathway^15^. In hypoxia, the activities of PHDs are inhibited, resulting in stabilized HIFα, which dimerizes with HIFβ, and activates the transcription of HIF-target genes such as glucose transporter 1 (GLUT1), phosphoglycerate kinase 1 (PGK1), and lactate dehydrogenase A (LDHA). PHDs comprise three isoforms, PHD1, PHD2 and PHD3^12^,^15^. Previous studies reported that among the three isoforms, PHD2, not PHD1 and PHD3, is a major negative regulator for vascular growth in adult mice^15^. When PHD2 is inactivated by hypoxia, accumulation of HIF-1α leads to the induction of vascular endothelial growth factor (VEGF) which enhances the growth of vascular beds. Considering that PT cells are enlarged in obesity and are easily damaged by hypoxia, we hypothesized that cellular adaptive processes are crucial for the pathophysiology of obesity-induced renal injury.

In this study, we used high-fat-diet (HFD)-fed C57BL/6J background mice as an obese mice model. Though this model is characterized by increased plasma free fatty acids (FFA) and insulin levels with impaired glucose tolerance, they did not show frank hyperglycemia. This model is relevant to study obesity-related nephropathy^3^. We observed renal hypoxia with perivascular rarefaction and PT enlargement in HFD-induced obese mice. Even though kidney tissue became hypoxic in HFD model, as shown by pimonidazole positive staining, hypoxic responses in HFD model appeared dysfunctional. Some of the HIF-1α target mRNAs including *Vegfa*, *Slc2a1*, *Pgk1*, and *Ldha* were not upregulated in response to hypoxia, indicating a lack of HIF-1α activation. By using tamoxifen (Tam)-inducible PT-specific *Phd2* knockout mice, we demonstrated that deletion of *Phd2* in PT cells mitigated the renal damage, by ameliorating the peritubular capillary rarefaction and tissue ischemia. Our data suggest that the inhibition of PHD2 in PT cells is a potential therapeutic strategy against obesity-induced kidney disease.

## Results

### Renal morphological changes and the hypoxic state in HFD-fed mice

**Figure 1:**
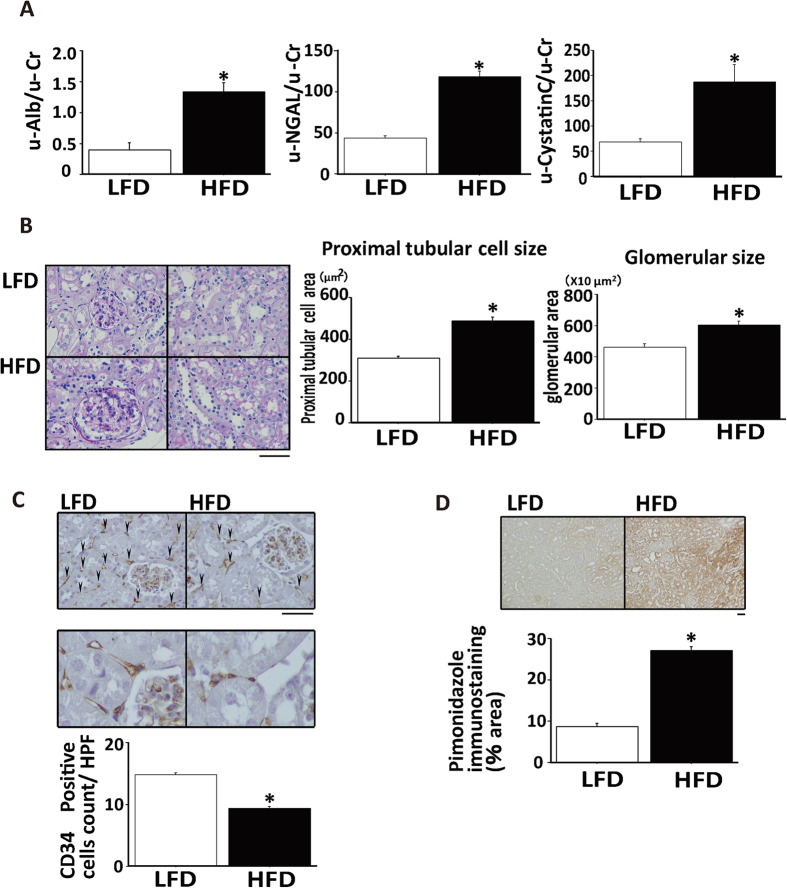
Renal injury and histological changes in obese mice. (**A**) Urinary excretion of albumin (u-Alb, left panel), neutrophil gelatinase-associated lipocalin (u-NGAL, middle panel) and cystatin C (right panel). (**B**) Representative pathology of kidneys of mice fed high fat diet (HFD) and low fat diet (LFD). Scale bar is 50 μm. Average areas of proximal tubule (PT) cells and glomeruli are shown in the right panel. (**C**) Immunostaining for CD34 (upper panel) in a low power field (LPF, upper pictures) and a high power field that focused on CD34+ cells (HPF, lower pictures). Lower bar graph shows the counts of CD34-positive cells reflecting the density of peritubular capillaries. Scale bar is 50 μm. (**D**) Immunostaining for pimonidazole (upper panel) and measurement of pimonidazole-positive areas, indicating a hypoxic state (lower panel). Scale bar is 50 μm. *p < 0.05 vs. LFD-fed mice, n = 8.

In mice fed HFD, body weight increased but blood pressure did not change (Table 1). Though fasting blood glucose did not differ between the two groups, serum levels of triglycerides, FFA, and insulin in fasted mice were higher in HFD-fed mice (Table 1). HFD-induced obese mice exhibited renal damage as indicated by albuminuria and excretion of the proximal tubular injury markers, neutrophil gelatinase-associated lipocalin (NGAL) and cystatin C (Fig. 1A) though serum creatinine levels were not changed (Table 1). These biochemical data were consistent with renal damage in obesity^5^. In histology, kidneys from HFD-fed mice exhibited marked mesangial hypercellularity and enlarged glomerular size. In addition, PT cellular size was increased in mice fed HFD, compared with in those fed LFD (Fig. 1B). These hypertrophic changes suggested a spatial sparse of peritubular capillary (PTC) beds. CD34-positive cell counts revealed that PTC density in the kidney was significantly decreased in HFD compared with LFD-fed mice (Fig. 1C). Because of PT enlargement and PTC rarefaction, we hypothesized that HFD-fed mice had PT hypoxia and performed pimonidazole staining to examine this alteration. Compared with LFD-fed mice, HFD-fed mice showed significantly increased pimonidazole-positive areas (Fig. 1D).

### Hypoxic response in kidneys of HFD-fed mice

**Figure 2:**
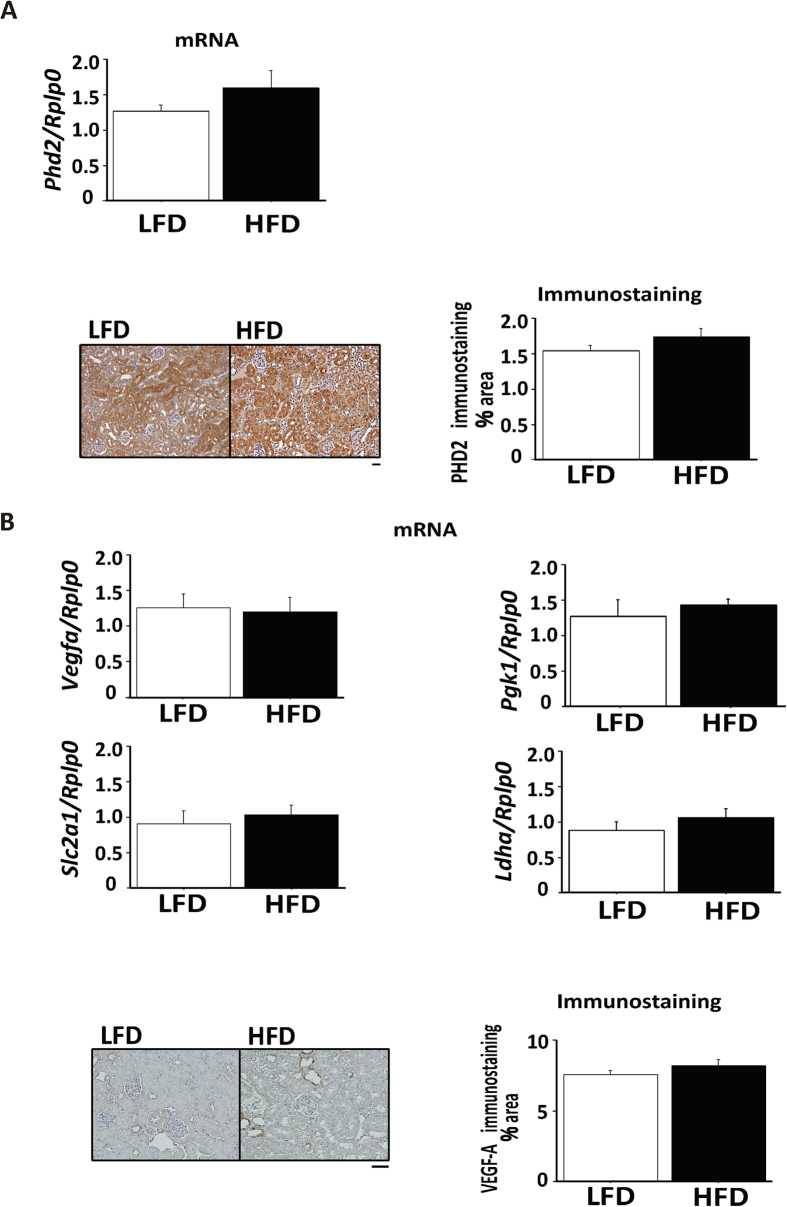
Lack of a hypoxic response in kidneys of HFD-fed mice. (**A**) mRNA expression of PHD2 in high fat diet (HFD) and low fat diet (LFD) fed mice. Bar graph represents the quantification of immunostained areas. Scale bar indicates 50 μm. (**B**) mRNA expression of *Vegfa, Slc2a1*, *Pgk1*, and *Ldha* in HFD and LFD-fed mice. Bar graph represents the quantification of immunostained areas. Scale bar is 50 μm.

To confirm molecular changes responsive to hypoxia, expressions of the downstream hypoxia-responsive molecules, including PHD2 and VEGF, were measured. Though the pimonidazole-positive areas in HFD-fed mice were significantly larger than in LFD-fed mice, expressions of either PHD2 (Fig. 2A) or genes downstream to PHD, including, *Vegfa* (VEGF-A), *Pgk1* (PGK1), *Slc2a1* (Glut-1), and *Ldha* (LDHA) (Fig. 2B) were not different between mice fed two diets. These data indicated that, in obese mice, renal tissues showed abnormal hypoxic responses.

### Generation of tamoxifen-inducible PT-specific PHD2 knockout mice

**Figure 3:**
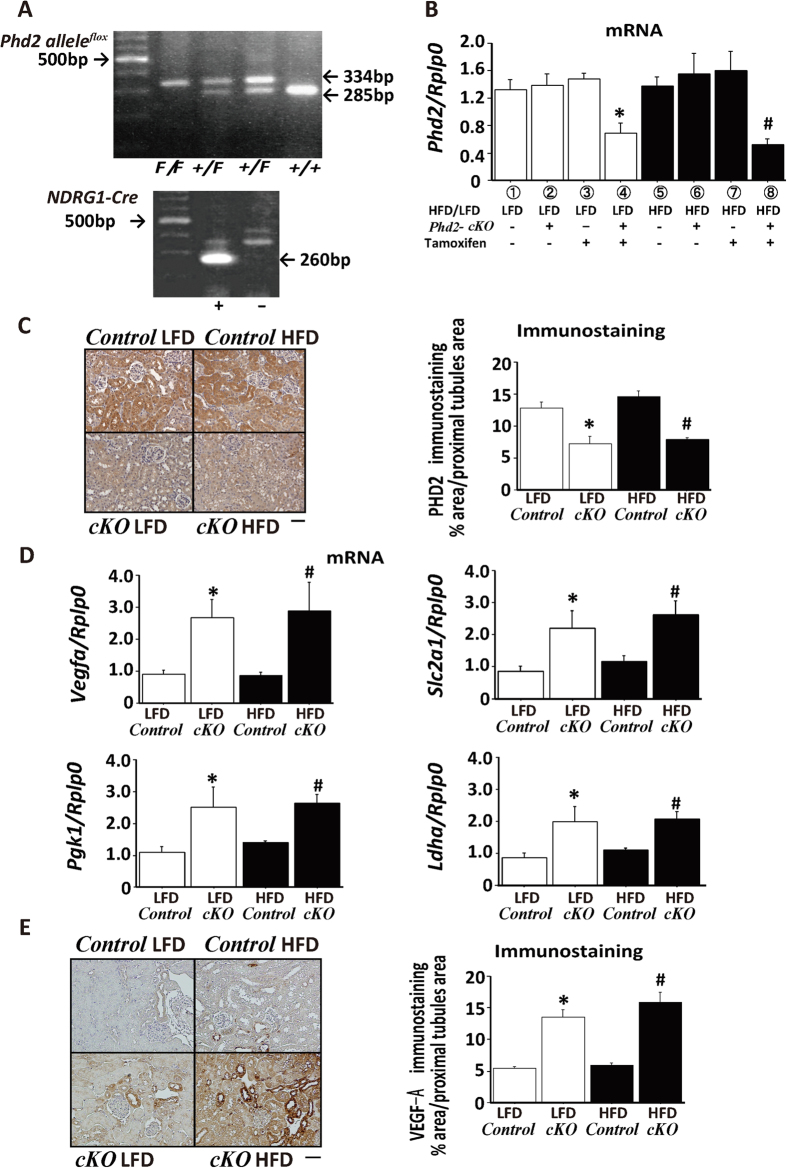
Tamoxifen-inducible proximal tubule-specific PHD2 knockout mice. (**A**) Representative results of genotyping of *Phd2 allele* (upper panel) and *Ndrg1-Cre* (lower panel) mice. PCR products are indicated by arrows. (**B**) Real-time RT-PCR analysis of mRNA expression of *Phd2* in kidney samples from each experimental group. Groups 1–4 represent low fat diet (LFD) and groups 5–8 represent high fat diet (HFD)-fed mice. Groups 2, 4, 6 and 8 represent mice harboring both *Phd2*^*F/F*^ and *Ndrg1-Cre* genes. Groups 3, 4, 7 and 8 represent mice treated with tamoxifen (Tam). *p < 0.05 vs. group 1, ^#^p < 0.05 vs. group 5. (**C**) Representative immunostaining for PHD2 (left panel) and quantitation of immunostained areas (right panel) in *Control* or *Phd2-cKO* mice fed with LFD or HFD. (**D**) mRNA expression of *Vegfa, Slc2a1*, *Pgk1*, and *Ldha* in *Control* or *Phd2-cKO* mice fed with LFD or HFD. (**E**) Representative immunostaining for VEGF-A (left panel) and quantitation of immunostained areas (right panel). *p < 0.05 vs. *Control* LFD, ^#^p < 0.05 vs. *Control* HFD. Scale bar indicates 50 μm.

To explore the role of an impaired hypoxic response in obese mice, we created Tam-inducible PT-specific *Phd2* knockout mice (*Phd2*-*cKO*) by crossing *Phd2*-floxed mice (*Phd2*^*F/F*^)^16^ with knock-in mice harboring Tam-inducible *Cre-recombinase* gene driven by a PT-specific N-myc downstream regulated gene 1 (*Ndrg1*) promoter^17^ (Fig. 3A). With Tam treatment, PHD2 expression was significantly decreased in the proximal tubules of *Phd2-cKO* mice on either diet group, confirming that *Phd2*-inactivation by the Cre-loxP system was effective in *Phd2-cKO* mice (Fig. 3B, columns 4 and 8). Tam treatment had no effect on PHD2 expression in *Control* mice (Fig. 3B, comparing columns 1 and 3 and columns 5 and 7). Immunostaining revealed that PHD2 expression was markedly decreased in Tam-treated *Phd2-cKO* mice (Fig. 3C). Expressions of downstream genes, *Vegfa* (VEGF-A), *Pgk1* (PGK1), *Slc2a1* (Glut-1), and *Ldha* (LDHA), were increased in *Phd2-cKO* mice, suggesting that hypoxia responsible gene downstream of PHD2 was subsequently induced by inactivating PHD2 (Fig. 3D). Immunostaining also showed the increase in VEGF-A expression in PT of *Phd2-cKO* mice (Fig. 3E). These data indicated that the hypoxic responses are functionally activated in *Phd2-cKO* mice on HFD as in those on LFD.

### Amelioration of the hypoxic condition by restoration of PTC in Tam-inducible PT-specific *Phd2-cKO* mice

**Figure 4:**
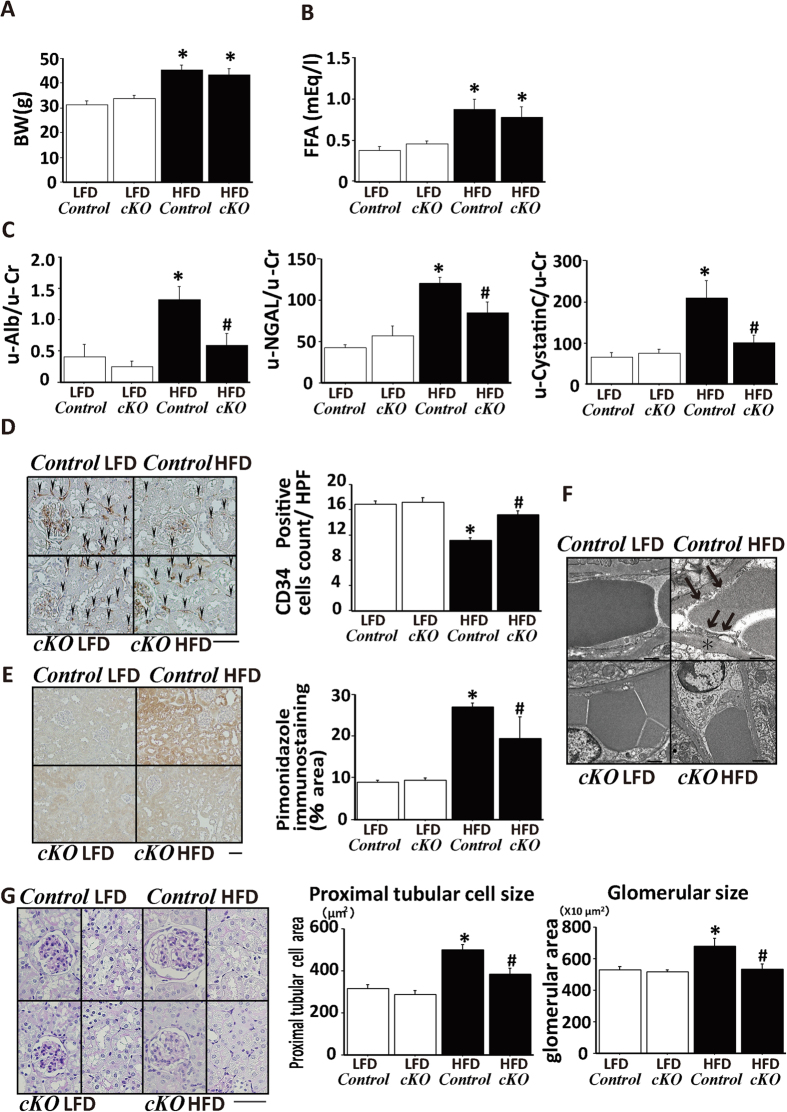
Phenotypes of PHD2 conditional knockout mice fed HFD or LFD. Body weight (**A**) serum concentrations of free fatty acids (**B**, FFA) in the four experimental groups. *Phd2-cKO*; tamoxifen (Tam)-inducible PT-specific *Phd2* knockout mice, *Control*; littermate control mice, LFD; low fat diet, HFD; high fat diet. (**C**) Urinary excretion of albumin (u-alb), neutrophil gelatinase-associated lipocalin (u-NGAL) and cystatin C in the four experimental groups. *p < 0.05 vs. *Control* LFD; ^#^p < 0.05 vs. *Control* HFD, n = 8. (**D**) CD34 immunostaining to assess peritubular capillary density. Bar graph shows the number of CD34-positive cells in a high power field (HPF). Scale bar is 50 μm. *p < 0.05 vs. *Control* LFD; ^#^p < 0.05 vs. *Control* HFD, n = 8. (**E**) Immunostaining for pimonidazole to detect hypoxic tissue. Bar graph shows the quantitation of pimonidazole-positive areas. Scale bar is 50 μm. *p < 0.05 vs. *Control* LFD; ^#^p < 0.05 vs. *Control* HFD, n = 8. (**F**) Representative findings of PTC in EM. In HFD mice, endothelial cells of PTC was enlarged, basement membrane was thickening (*), slit structures collapsed (black arrows), and lumen of PTC was narrowing, which were ameliorated in *Phd2-cKO* HFD mice. (**G**) Representative pathology of the kidneys, including glomeruli and tubulointerstitial lesions, from each experimental group. Bar graph shows the average areas of proximal tubule cells and glomeruli. *p < 0.05 vs. *Control* LFD; ^#^p < 0.05 vs. *Control* HFD, n = 8.

Because it is hypothesized that hypoxic response does not work properly, thereby contributing to development of renal pathological lesions in obese mice, we examined effects of PHD2-suppression on renal damage using the Tam-inducible PT-specific *Phd2-cKO* mice. HFD increased body weights of both *Control* and *Phd2-cKO* mice, with no weight difference between *Phd2-cKO* and *Control* mice on either diet group (Fig. 4A). HFD increased serum FFA similarly in *Phd2-cKO* and *Control* mice (Fig. 4B). Albuminuria, the marker for glomerular damages, as well as the markers for proximal tubular injury including urinary excretion of NGAL and cystatin C were higher in *Control* HFD mice compared with *Control* LFD mice. These HFD-dependent changes were attenuated in *Phd2-cKO* mice with Tam treatment (Fig. 4C). To explore the mechanism whereby *Phd2* gene silencing ameliorated renal damages in HFD-induced obese mice, we examined the tissue ischemic state. The number of CD34-positive cells was decreased in *Control* HFD mice compared with *Control* LFD mice. This capillary loss was restored in *Phd2-cKO* HFD mice with Tam treatment (Fig. 4D). Consistent with these results, the pimonidazole-positive area was increased in *Control* HFD mice. However, in *Phd2-cKO* HFD mice, pimonidazole-positive area was smaller, nearly the same as in *Control* LFD mice (Fig. 4E). In addition to these results, electron microscope showed PTC injuries in *Control* HFD mice. In HFD mice, endothelial cells of PTC was enlarged, basement membrane was thickening, slit structures collapsed, and lumen of PTC was narrowing, which were ameliorated in *Phd2-cKO* HFD mice (Fig. 4F). This indicated that, in *Phd2-cKO* mice, ischemia was ameliorated in the HFD-induced obese state. The histological abnormalities in *Control* HFD mice, including glomerulomegaly and enlarged PT cellular size, were completely prevented in *Phd2-cKO* HFD mice with Tam treatment, suggesting that the early intervention could suppress the progression to the renal pathological changes of both tubular and glomerular lesions in the HFD-induced obese state (Fig. 4G).

### Dysregulation of hypoxic response induced by FFA

**Figure 5:**
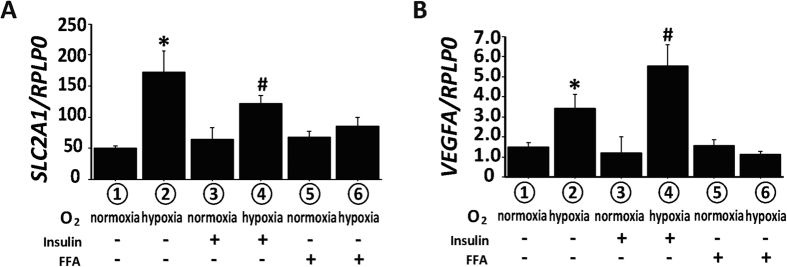
Effects of insulin and free fatty acids on the hypoxic response of HK-2 cells. The mRNA expression of *SLC2A1* (Glut1: glucose transporter1) (**A**) and *VEGFA* (**B**). HK-2 cells were subjected to normoxic (normoxia) or hypoxic (hypoxia) culture conditions and pretreated with vehicle (groups 1 and 2), insulin (groups 3 and 4) or free fatty acids (FFA) (groups 5 and 6). Each bar graph represents the results of six experiments. *p < 0.05 vs. group 1, ^#^p < 0.05 vs. group 3, n = 8.

To further examine the mechanism for the impaired hypoxic response observed in HFD-fed obese mice, a human PT cell line, HK-2 cells, were cultured in either normoxic or hypoxic (1% O_2_) condition to mimic kidney environment in HFD-induced obese mice. The expressions of hypoxia-responsible genes downstream of PHD2-HIF pathway including glucose transporter 1 (*GLUT1*) and *VEGFA* were increased under hypoxia (Fig. 5A,B, lanes 1 and 2). We examined the effects of insulin or FFA, both of which were systemically elevated in HFD-induced obesity (Table 1). Insulin did not affect the expression of *SLC2A1* (*GLUT1*. Fig. 5A, lanes 3 and 4) or *VEGFA* (Fig. 5B, lanes 3 and 4). However, FFA dampened the upregulation of these genes by hypoxia (Fig. 5A,B, lanes 5 and 6).

## Discussion

Obesity and metabolic syndrome are complicated with renal damages in which multiple factors, including systemic hypertension, dyslipidemia and abnormal glucose metabolism are involved. We previously reported obesity-induced histological changes in the kidney, including glomerular hypercellularity, macrophage infiltration and vacuolization in PT^5^. In the present study, we demonstrated that hypoxic tissue insults are present in obese kidney, which is caused by PTC rarefaction through the impaired hypoxic response. We also demonstrated that the restoration of oxygen supply by induced PT-specific PHD2 deletion mitigated not only in tubular damages but also glomerular damages. This restoration also ameliorated renal histological changes including both tubular and glomerular hypertrophy. Our data provide the evidence for a crucial role of an improper hypoxic response in PT to hypoxia in the development of renal lesions of both tubules and glomeruli in the obese condition. It is also demonstrated how the aberrant PT function propagate to interstitial vascular disorganization or glomerular damages to establish the obesity-induced renal damages.

In response to hypoxic stress, cellular adaptive processes are regulated by HIF-1 and HIF-2 in various tissues including kidney^18^. Under hypoxia, PHD2 is inactivated since its activity is dependent on oxygen molecules, and the hydroxylation of HIF-1α is inhibited, causing HIF-1α to accumulate and inducing genes downstream to HIF, including *Vegfa*, *Pgk1*, *Slc2a1*, and *Ldha*. However, in the obese mice, even under hypoxic conditions, the expression of these hypoxia-responsible genes were not induced (Fig. 2), suggesting the impaired hypoxic response. Using an inducible gene-engineered mouse model, we have now shown that these lesions were almost fully eliminated by PHD2 downregulation in PT, suggesting that obesity-induced renal damage is attributed mainly to the lack of an inactivation of PHD2 in PT. This reactivation of HIF-VEGF pathway by deletion of the *Phd2* gene in PT increased number of PTCs and subsequently prevented hypoxic renal injuries. It was recently reported that tubulo-vascular crosstalk involving VEGF is essential to maintain PTC networks in the kidney^19^,^20^. Our results support this concept, providing new evidence for intercellular communication between PT cells and PTC cells and for the pathological relevance of this communication in obesity-induced kidney disease. Moreover, we further implicated FFA as one of the candidate factors for impairing the hypoxic response of PT cells although the detailed mechanism has not been elucidated. It was demonstrated that the activation of nuclear receptor, peroxisome proliferator-activated receptors γ (PPARγ) induces PHD up-regulation in adipocytes^21^. Since certain kinds of FFA and their derivatives including palmitate and oleate that were used in the present *in vitro* study have been shown to activate PPARγ^22^,^23^. HFD-induced increase in serum FFA concentration might maintain the PHD2 activity and inhibit the inactivation of PHD2 in spite of hypoxic condition. Our data provide mechanistic clues that dyslipidemia is closely associated with CKD progression^24^,^25^ and controlling hyperlipidemia could augment HIF-VEGF signaling and correct an impaired hypoxic response.

The cellular enlargement of PT, described as histological findings in obesity^4^,^5^, would be expected to contribute to the tissue hypoxic condition through the rarefaction of PTCs. However, the mechanism of cellular enlargement of PT is not clear. In previous reports, a variety of factors, including insulin or tubular cell autophagy, could also induce tubular cell enlargement. Insulin is a well-known inducer of cellular hypertrophy by activating mammalian-target of rapamycin (mTOR)/S6 kinase pathway^26^,^27^. It has been also reported that autophagy-deficient renal tubular cells in *Atg6*-deficient mice accumulated deformed mitochondria and cytoplasmic inclusions, leading to cellular hypertrophy^28^ and that, in obesity-induced renal damages, autophagy insufficiency in PT cells exacerbates proteinuria-induced tubulointerstitial lesions^29^,^30^. In our study, PT enlargement was alleviated in HFD-fed *Phd2-cKO* mice, supporting the idea that tubular hypertrophy in obesity-induced kidney injury may be caused by insufficient hypoxic response in PT. Cellular hypertrophy is often associated with cell growth arrest^31^,^32^ and hypoxia is known to be one environmental factor that halts the cell cycle and cell proliferation^33^,^34^. Hypoxia was reported to upregulate the cyclin dependent kinase inhibitors p21^Cip1^ and p27^Kip1^, which block cell cycle progression^35^. Hypoxia may also induce cell cycle arrest by inhibiting c-Myc transcriptional activity^36^. Another molecular mechanism for the relationship between hypoxic condition and cellular hypertrophy is the AMP-activated protein kinase (AMPK) signaling pathway that is activated by increased ratio cellular AMP/ATP ratio in hypoxic condition^37^. AMPK activation attenuated cellular hypertrophy^38^,^39^ by inhibiting protein synthesis through the mTOR pathway^40^. Using models for renal disease, Li *et al.* showed that HIF-1α and AMPK were linked at a molecular level during the response to hypoxic stress in the pathophysiology of CKD. AMPK activation was decreased in the subtotal nephrectomy model and was markedly restored by HIF-1α activation^41^. In our study, we speculate that HIF-1α did not function normally and AMPK may not have been fully restored by HIF-1α to facilitate cellular adaptation to hypoxia, resulting in cellular hypertrophy.

In the present study, we observed in detail PTC injuries in EM. Of note, in HFD mice, endothelial cells of PTC was enlarged, basement membrane was thickening, slit structures collapsed, and lumen of PTC was narrowing, which are new findings in obesity induced renal injuries and firstly reported. In addition to PTC rarefaction, these PTC injuries may contribute to hypoxic tissue insults in obese kidney. Although previous studies of obesity induced renal histological changes in human focused primarily on the glomerular area and obesity-related glomerulopathy has been characterized by glomerulomegaly with or without focal segmental glomerulosclerosis^4^, whether new findings in the present study are observed also in human needs to be evaluated in the future study.

One salient finding in our study is that albuminuria and glomerular hypertrophy in histology were also remitted by improving the hypoxic condition through an intervention affecting PHD2. We speculate that the activation of HIF-VEGF pathway by PHD2 deletion in PT might lead to the restoration of post-glomerular PTC network, resulting in the reduction of glomerular afterload and the amelioration of glomerular hypertension and hypertrophy. These alterations cumulated in the reduction of albuminuria in obese mice. Alternatively, this result is related to a novel pathological link between tubular lesion and glomerular lesion in diabetic nephropathy we recently reported^42^. In this study, the decreased expression of NAD^+^ -dependent deacetylase Sirt1 in PT initiates diabetic albuminuria through the downregulation of Sirt1 and the upregulation of claudin-1, a tight junction component in podocytes. The present study provides evidence for this renal tubule–glomeruli communication and through this communication, the recovery from tubular damages leads to the recovery from glomerular damages and albuminuria. Therefore, the manipulation targeting PHD2-dependent hypoxic response has a promise for inhibiting the progression of glomerular damage in obese-induced kidney disease.

Several limitations of the present study merit comments. The present study demonstrated the results with short diet duration and there may be different findings with longer duration. In addition, it is difficult to show the activity of PHD2 *in vivo* and also to show the expression of HIF-1α by lacking of high quality antibodies. Instead, we have demonstrated that hypoxic state is detected by pimonidazole staining and shown the expression of mRNAs or proteins of downstream genes, but not HIF-1α. Although it has been described that the increase in glomerular basement membrane thickness may be seen after the onset of diabetes, we have firstly reported that basement membrane of tubules is already thickened in obesity before the onset of diabetes. However, it is needed to make sure of whether the same histological changes are observed in obesity in human. Further studies are required to clarify these limitations.

In conclusion, this is the first study to demonstrate that a hypoxic condition due to inadequate hypoxic response is a pathophysiological effect associated with obesity-induced renal injury. Normalization of hypoxic response by the downregulation of PHD2 in PT ameliorated hypoxic damage not only of PT lesions also in glomerular lesions. An early intervention targeting PHD2, specifically in the proximal area, may represent a novel therapeutic strategy against the progression of obesity-induced kidney injury.

## Materials and Methods

### Animal 1: HFD-fed wildtype mice

Eight-wk-old male C57BL/6J mice (Clea Japan, Tokyo, Japan) weighing 20 ± 1 g were housed in a temperature- and light (22 ± 1 °C; 12-hour light/dark cycle)-controlled room with *ad libitum* access to tap water and standard mouse chow. Animals were fed LFD, (10% lard, Research Diets Inc. New Brunswick, NJ; n = 8) or HFD (60% lard, Research Diets Inc.; n = 8) during the 12-wk experimental protocol as described before^5^.

### Animal 2: Inducible PT-specific PHD2 knockout mice

We generated PT-specific conditional *Phd2* knockout (*Phd2-cKO*) mice by crossing the *Phd2*^*F/F*^ mice from Dr. Yoji Andrew Minamishima (Keio University)^16^ and the *Ndrg1-Cre*^*ERT2/*+^ mice with a PT-specific *Ndrg1* gene promoter from Prof. Motoko Yanagita (Kyoto University)^17^. To obtain *Phd2-cKO* mice, *Phd2*^*F/F*^ mice were crossed with *Ndrg1-Cre* mice to generate *Phd2*^+*/F*^*; Ndrg1-Cre* mice. *Phd2*^+*/F*^*; Ndrg1-Cre* mice were then crossed with *Phd2*^+*/F*^ mice to generate *Phd2*^*F/F*^*; Ndrg1-Cre* (*Phd2-cKO*) mice and *Phd2*^+/+^; *Ndrg1-Cre* (*Control*) as littermate controls. Genomic DNA was isolated from tail biopsies at 4 week of age using a DNeasy kit (Qiagen Inc., Valencia, CA, USA) and genomic DNA samples were screened by polymerase chain reaction using the transgene-specific oligonucleotide primers shown in Table 2. The primers used to amplify the *Phd2*^*F/F*^ allele are also shown in Table 2. The *Cre* gene was designed to be specifically activated by the estrogen receptor (ER) agonist, tamoxifen (Tam). PT-specific conditional *Phd2* knockout mice (*Phd2-cKO*) and their control littermates (*Control*) were maintained on LFD (n = 8) or HFD (n = 8) from 8 to 20 wk of age. Mice were divided into 8 groups; (1) LFD-fed *Control* mice without Tam injection (sunflower oil vehicle injected) (*Control* LFD/Tam−); (2) LFD-fed *Phd2-cK*O mice without Tam injection (*Phd2-cKO* LFD/Tam−); (3) LFD-fed *Control* mice with Tam injection (*Control* LFD/Tam+); (4) LFD-fed *Phd2-cKO* mice with Tam injection (*Phd2-cKO* LFD/Tam+); (5) HFD-fed *Control* mice without Tam injection (*Control* HFD/Tam−); (6) HFD-fed *Phd2-cKO* mice without Tam injection (*Phd2-cKO* HFD/Tam−); (7) HFD-fed *Control* mice with Tam injection (*Control* HFD/Tam+); and (8) HFD-fed *Phd2-cKO* mice with Tam injection (*Phd2-cKO* HFD/Tam+). At 8 wk of age, mice received 1 mg of tamoxifen (Sigma Aldrich, St. Louis, MO, USA) by intraperitoneal injection for 5 consecutive days as described previously^43^. To visualize renal hypoxia, pimonidazole (60 mg/kg) was administered by intraperitoneal injection 90 min before sacrifice^44^. Mice were kept on HFD or LFD and sacrificed on week 12 as described in the Animal-1 experiment.

### Blood and urine analyses

Serum concentrations of insulin and free fatty acids (FFA) were measured with LabAssay^®^ (Wako Pure Chemical Industries, Ltd., Osaka, Japan). Urinary albumin was measured by Albuwell M (Exocell Inc., Philadelphia, PA, USA) and neutrophil-associated lipocalin (NGAL) and cystatin C were measured by ELISA kits (Quantikine, R&D Systems, MN, USA).

### Morphological examination and Immunohistochemistry

Kidney sections (5 μm) were stained with periodic acid-Schiff’s stain^45^. Immunohistochemical staining was performed using primary antibodies against CD34 (ab81289), PHD2 (ab109088) and vascular endothelial growth factor (VEGF) (ab46154) (Abcam, Cambridge, MA, USA) and an anti-pimonidazole antibody (Hypoxyprobe™-1 Omni Kit, Burlington, MA, USA). Horseradish peroxidase-conjugated anti-rabbit IgG antibodies (Dako, Glostrup, Denmark) were used as secondary antibodies. Staining was visualized with a diaminobenzidine (DAB) chromogen, followed by counterstaining with hematoxylin. The extent of histochemical and immunohistochemical staining were quantified using computer-assisted image analysis^46^. Sections incubated with normal rabbit serum instead of the primary antiserum served as negative controls. Fifty glomeruli and fifty proximal tubular cells were counted in one kidney section from each mouse. The areas of the glomeruli and PT were measured by image analysis of high magnification photographs. Immunostaining was assessed at 100x, 200x or 400x magnification using 20 randomly selected fields for each mouse. These morphological evaluations were conducted in a blinded manner by two independent observers.

### Transmission electron microscopic (TEM) analysis

TEM analysis was performed as previously reported^47^. Briefly, the kidneys were dissected out without any perfusion and were fixed with 2.5% glutaraldehyde in 100 mM phosphate buffer (pH 7.4) for 12 hours at 4 °C. After the 2 hours of post-fixation with 1% osmium tetroxide, dehydrated through ethanol, acetone, QY1, and embedded into the epon. For 72 hours of complete polymerization with pure epon, ultrathin sections were prepared with a thickness of 70 nm and stained with uranyl acetate and lead citrate for 10 minutes. The sections were observed under a transmission electron microscope (JEOL model 1400plus).

### Cell culture

The human renal proximal tubular cell line, HK-2 cells (American Type Culture Collection, Rockville, MD, USA) were expanded in Dulbecco’s modified Eagle’s medium nutrient mixture F-12 (GIBCO Life Technologies, Foster City, CA, USA) with 10% bovine serum (FBS) supplemented with 50 U/mL penicillin and 50 *μ*g/mL streptomycin in a CO_2_ incubator (95% air and 5% CO_2_, 37 °C and 95% humidity; defined as normoxia).

### Hypoxic exposure and stimulation by insulin or FFA

Early passage (passages 3–4) HK-2 cells were grown to 80% confluence and made quiescent by serum starvation for 24 h. Cells were then exposed to hypoxia (1% O_2_, 5% CO_2_, 94% nitrogen gas) for 24 h in a hypoxia workstation (Hirasawa Works, Tokyo, Japan) or remained in the CO_2_ incubator used for routine culture (normoxia). An oxygen sensor was used to ensure that the oxygen concentration inside the workstation was maintained at 1% throughout the experiments (MC-8G-S, Iijima electronics corporation, Aichi, Japan). Cells were further supplemented with 1 μM human insulin (Sigma Aldrich) or 0.3 mM FFA. The FFA stock solution consisted of 6.35 mM sodium palmitate (Sigma Aldrich) and 12.7 mM sodium oleate (Sigma Aldrich) in FFA-free bovine serum albumin (BSA) (1.8 mM) as previously described^48^,^49^. Cells were divided into six groups: (1) cells without insulin or FFA in normoxia (normal O_2_ concentration of 20%), (2) cells without insulin or FFA in hypoxia, (3) cells with insulin in normoxia, (4) cells with insulin in hypoxia, (5) cells with FFA in normoxia and (6) cells with FFA in hypoxia. After incubation for 24 h, cells were collected using TRIzol reagent (Invitrogen, Carlsbad, CA) to perform real-time RT-PCR.

### Real-time RT-PCR

Equal amount (1 μg) of total RNA from each sample was subjected to reverse transcription in a 20 μL reaction mixture containing random primers and Superscript II enzyme (Invitrogen). Real-time PCR was performed using an ABI Step One Plus sequence detector (Applied Biosystems, Foster City, CA)^50^. mRNA levels were normalized to those of *RPLP0* (*36B4*) genes. The sequences of primers used are shown in Table 3.

### Statistical Analysis

Data are expressed as means ± standard error of mean. One-way analysis of variance was used to determine significant differences among groups. In the overall analysis of variance, the Kruskal–Wallis test for multiple comparisons was used to assess individual group differences. *P* < 0.05 was considered statistically significant.

### Ethics Statement

This study was performed in accordance with the Institutional Guidelines on Animal Experimentation at Keio University. All methods were carried out in accordance with the guidelines for animal experiments of the Ministry of Education, Culture, Sports, Science and Technology, Japan. The experimental protocols were approved by the Animal Care and Experimentation Committee in Keio University (ID; 09119-(3)). All surgery was performed under sodium pentobarbital anesthesia by intraperitoneal injection, and all efforts were made to minimize suffering. At the end of the experiments, the mice were euthanized by intraperitoneal injection of sodium pentobarbital.

## Additional Information

**How to cite this article**: Futatsugi, K. *et al.* Obesity-induced kidney injury is attenuated by amelioration of aberrant PHD2 activation in proximal tubules. *Sci. Rep.* **6**, 36533; doi: 10.1038/srep36533 (2016).

**Publisher’s note**: Springer Nature remains neutral with regard to jurisdictional claims in published maps and institutional affiliations.

## Figures and Tables

**Table 1:** The baseline characteristics of LFD-fed or HFD-fed mice.

	**LFD-fed mice**	**HFD-fed mice**
Body Weight (g)	32.9 ± 0.811	40.4 ± 1.499**
Systolic blood pressure (mmHg)	111 ± 2.07	116 ± 2.19
Diastolic blood pressure (mmHg)	64.9 ± 1.83	66.8 ± 1.11
Total cholesterol (mg/dl)	74.8 ± 2.35	214 ± 11.9**
Free tatty acid (mEq/l)	0.395 ± 0.0941	1.06 ± 0.132*
Fasting blood glucose (mg/dl)	116 ± 26	126 ± 22
Fasting insulin (ng/ml)	4.39 ± 1.82	10.3 ± 1.84*
Serum Creatinine (mg/dl)	0.797 ± 0.204	0.967 ± 0.322

Data were presented with mean ± SEM, **p < 0.01 vs. LFD-fed mice, *p < 0.05 vs. LFD-fed mice, n = 8.

**Table 2:** Sequences of primers for genotyping.

*Ndrg1-Cre transgene*	Forward	GTGCCTGGCTAGAGATCCTG
	Reverse	AGAGACTTCAGGGTGCTGGA
*Phd2*-floxed allele	Forward	AGATGACCTCCCCAACTCTGCTAC
	Reverse	CAGTGTTCTGCCTCCATTTAT
*Phd2*-null allele	Forward	TCCATCCAGTCTGAGTTTCTTTCC
	Reverse	CAGTGTTCTGCCTCCATTTAT

*Ndrg1*; N-myc downstream regulated gene 1, *Phd2*; prolyl-hydroxylase domain-containing protein 2.

**Table 3:** Sequences of each primer for real-time RT-PCR.

mouse *Phd2*	Forward	GCCCAGTTTGCTGACATTGAAC
	Reverse	CCCTCACACCTTTCTCACCTGTTAG
mouse *Vegfa*	Forward	GTGCACTGGACCCTGGCTTTA
	Reverse	GGTCTCAATCGGACGGCAGTA
mouse *Rplp0*	Forward	GGCCCTGCACTCTCGCTTTC
	Reverse	TGCCAGGACGCGCTTGT
mouse *Pgk1*	Forward	GATGAGGGTGGACTTCAAC
	Reverse	TAAGGACAACGGACTTGGC
mouse *Ldha*	Forward	ACAGTTGTTGGGGTTGGTGC
	Reverse	CGCAGTTACACAGTAGTCTTTG
human *VEGFA*	Forward	AGCCTTGCCTTGCTGCTCTA
	Reverse	GTGCTGGCCTTGGTGAGG
human *SLC2A1*	Forward	GGCCAAGAGTGTGCTAAAGAA
	Reverse	ACAGCGTTGATGCCAGACAG
human *RPLP0*	Forward	GCAATGTTGCCAGTGTCTGT
	Reverse	GCCTTGACCTTTTCAGCAAG

*Vegfa*; vascular endothelial growth factor A, *SLC2A1*; glucose transporter 1 (GLUT1), *Pgk1*; Phosphoglycerate kinase 1, *Ldha*; Lactate dehydrogenase A, *Rplp0*; 60S acidic ribosomal protein P0 (36B4).
